# Novel Pink Bollworm Resistance to the Bt Toxin Cry 1Ac: Effects on Mating, Oviposition, Larval Development and Survival

**DOI:** 10.1673/031.009.2401

**Published:** 2009-05-22

**Authors:** J.A. Fabrick, L. Forlow Jech, T. J. Henneberry

**Affiliations:** USDA-ARS, Arid Land Agricultural Research Center, 21881 North Cardon Lane, Maricopa, AZ 85238

**Keywords:** *Pectinophora gossypiella*, *Bacillus thuringiensis*, transgenic cotton

## Abstract

Bt cotton plants are genetically engineered to produce insecticidal toxins from the *Bacillus thuringiensis* (Bt) Berliner (Bacillales: Bacillaceae) bacterium and target key lepidopteran pests. In all previous strains of pink bollworm, *Pectinophora gossypiella* (Saunders) (Lepidoptera: Gelechiidae) selected in the laboratory for resistance to insecticidal Cry1Ac toxin using an artificial diet containing the toxin, resistance to Cry1Ac and to Bt cotton is linked to three cadherin alleles (*r1*, *r2*, and *r3*). In contrast, the BG(4) pink bollworm strain was selected for resistance to Bt cotton by feeding larvae for four days in each of 42 generations on bolls of ‘NuCOTN33B®’ that expressed Cry1Ac toxin. After additional selection for eleven generations on Cry1Ac-incorporated diet, the susceptibility to Cry1Ac, fecundity, egg viability, and mating of this strain (Bt4R) was compared with the unselected Cry1Ac-susceptible parent strain. Some larvae of the Bt4R strain survived on diet containing ≥ 10 µg Cry1Ac per milliliter artificial diet, but none survived on transgenic cotton bolls. In contrast to strains selected exclusively on Cry1Ac diet, some survival of progeny of reciprocal moth crosses of Bt4R resistant and Bt-susceptible strains occurred on Cry1Ac-treated diet, suggesting differences in levels of dominance. The Bt4R resistant strain does not have the *r1, r2*, or *r3* mutant cadherin genes as do all previous strains of pink bollworm selected on Cry1Ac-treated artificial diet. The combined results suggest a mechanism of resistance to Cry1Ac that is different from previously described cadherin mutations.

## Introduction

Transgenic cotton with the *Bacillus thuringiensis* (Bt) Berliner (Bacillales: Bacillaceae) gene or genes producing proteins toxic to the pink bollworm, *Pectinophora gossypiella* (Saunders) (Lepidoptera: Gelechiidae), and other lepidopterous pests have been grown commercially in Arizona since 1996 without loss in control efficacy (Tabashnik et al. 2004; Tabashnik et al. 2006). This has occurred irrespective of the predictions for Bt resistance development based on experience with insecticides (Mellon and Rissler 1998) and documented examples of resistance to Bt sprays in greenhouse and field populations of some lepidopterans (Janmaat and Myers 2003; Tabashnik 1994; Ferré and Van Rie 2002). Four *P. gossypiella* strains have been selected for resistance using Cry1Ac protein from MVP-II® formulation incorporated in artificial diet (Tabashnik et al. 2003; Tabashnik et al. 2004). Larvae of these diet-selected resistant *P. gossypiella* strains survive and develop to reproductive adults on bolls of commercial transgenic cottons expressing Cry1Ac (Tabashnik et al. 2004; Tabashnik et al. 2005a). Resistance to Cry1Ac in these *P. gossypiella* is controlled by one or a few loci with recessive autosomal inheritance and without maternal or sex-linkage (Liu et al. 2001; Tabashnik et al. 2002). Three cadherin alleles (*r1, r2*, and *r3*) are genetically-linked with resistance to Cry1Ac and Bt cotton in all previously described resistant *P. gossypiella* strains (Morin et al. 2003, Morin et al. 2004; Tabashnik et al. 2004, Tabashnik et al. 2005a). Selection for resistance to Bt does not always result in the ability of resistant insects to survive on their natural host plants (Tabashnik et al. 2003), indicating that selection to formulated toxin differs from that on transgenic plants and that additional factors may contribute to insect mortality.

In contrast to selection for resistance using Cry1Ac protein in artificial diet, BG(4) *P. gossypiella* larvae selected for resistance in laboratory studies from 2001 to 2005 by feeding on ‘NuCOTN33B®’ (Bt) cotton bolls did not produce Bt resistant larvae that survived on Bt cotton bolls (Henneberry et al. 2006). Some larvae of this selected strain, however, did survive at ≥10 µg Cry1Ac per milliliter artificial diet, which has been determined to be a discriminating concentration for *P. gossypiella* resistance in the laboratory (Tabashnik et al. 2003). Emergence, mating, and egg hatch were not significantly affected by selection, but fecundity was significantly reduced in BG(4). This suggests a cost of resistance similar to those reported for other *P. gossypiella* selected strains (Carrière et al. 2001a,b), although incomplete resistance (Carrière et al. 2006) or inbreeding depression could have contributed to such loss in fecundity.

Here, a *P. gossypiella* Bt4R strain was generated by further selecting the Cry1Ac-tolerant BG(4) strain on artificial diet containing Cry1Ac for 11 generations. Susceptibility was compared to Cry1Ac, fecundity, egg viability, and mating of the Bt4R strain with a Cry1Ac-susceptible strain (WCRL) and provide an initial comparison of resistance with known diet-selected *P. gossypiella* strains. Bt4R adults were analyzed for the presence of known cadherin resistance alleles. Finally, the survival and development between F1 progeny from reciprocal crossmating of the Bt4R resistant strain and a susceptible strain on Cry1Ac-incorporated diet was compared. The results suggest that the mechanism of resistance to Cry1Ac in Bt4R differs from previously characterized *P. gossypiella* strains.

## Materials and Methods

### Pink bollworm strains

Bt-susceptible *P. gossypiella* used in the study were from the Western Cotton Research Laboratory, Phoenix, AZ colony (designated as the WCRL strain) that has been reared continuously without selection on artificial diet (Bartlett and Wolf 1985) for more than 350 generations. A Cry1Ac-resistant strain [BG(4)] was developed by selection of over 3,000 individuals from the WCRL strain over 42 generations of four-day feeding on Bt cotton bolls that expressed Cry1Ac (Henneberry et al. 2006). Following the 4 day feeding period on Bt cotton bolls, surviving larvae were reared until adulthood on artificial diet without Bt toxin. The initial selection of BG(4) consisted of 220 neonate larvae placed on 44 cotton bolls for 4 days, of which 112 pupae survived and were used for subsequent selections. Approximately 50–100 individuals in each generation were selected on Bt cotton bolls. An additional *P. gossypiella* strain was selected on Bt cotton bolls for seven days [BG(7)]; however, the BG(7) strain was less fit than the BG(4) strain and was ultimately lost (Henneberry et al. 2006). A sub-colony of the BG(4) strain at generation 42 was established on artificial diet containing 10 µg of Cry1Ac toxin per milliliter diet and is designated as the Bt4R strain. Selection of Bt4R on Cry1Ac-treated diet resulted in sufficient adults to maintain the colony, ranging from 62 moths from initial selection to hundreds of adults in subsequent generations. This concentration of Bt toxin was chosen as previous experiments have identified it as diagnostic for identifying larvae that are homozygous for resistance (Tabashnik et al. 2002). Experiments on Bt4R began after 11 generations on Cry1Ac-treated diet.

For each generation of Bt4R on Cry1Ac diet, surviving pupae were held in 9.5 cm × 6.0 cm waxed cardboard mating-oviposition cages. Emerging adults were provided a 10% sucrose solution for food and paper towel pieces (2.5 cm^2^) were placed over screens to act as an oviposition substrate. Oviposition substrates with eggs were collected and placed in cardboard containers filled with approximately 100 g of 10 µg Cry1Ac per ml artificial diet to start the following generation. Larvae were reared at 26.7°C in constant temperature cabinets with a 14:10 L:D cycle. The WCRL strain was reared as described above, but on artificial diet without Cry1Ac toxin.

### Cry1Ac-treated diet

Cry1Ac-treated diet was supplied by the Extension Arthropod Resistance Management Laboratory (EARML) of the University of Arizona, Tucson, AZ. Cry1Ac toxin (**MVP-II®Bioinsecticide, Mycogen, San Diego, CA**) in stock solution was mixed into artificial diet in amounts necessary to create final concentrations ranging from 0 to 1000 µg Cry1Ac/mL of diet as described by Dennehy et al. (2004).

### Cry1Ac susceptibility

The susceptibility of the Bt4R and WCRL pink bollworm strains to Cry1Ac was determined using a modification of the survival method of Tabashnik et al. (2002). Approximately 2 g of artificial diet containing 0, 0.1, 0.3, 1, 10, 32 or 100 µg Cry1Ac/ml was placed in five 30 ml plastic cups. Five neonate larvae from each strain were placed in each of the cups using a fine brush. Larvae were reared at 26.7°C with 14:10 L:D. Live and dead insects were recorded after 21 days, when any fourth instar larvae, pupae, or adults were considered survivors. An additional dose-mortality experiment was performed on the Bt4R strain, as higher concentrations of Cry1Ac were needed to estimate the LC_50_. For the Bt4R strain only, newly hatched neonates (3 replicates of n = 30) were reared individually on 0, 1, 10, 100, and 1000 µg Cry1Ac per ml artificial diet in cups as described above and mortality was recorded after 21 days. LC_50_s were estimated by Probit analysis (PROC PROBIT) using SAS (SAS Institute 1985). The resistance ratio was calculated as the LC_50_ for Bt4R divided by the LC_50_ for WCRL.

### PCR analysis for known cadherin *r* alleles

DNA screening of *P. gossypiella* for the presence of known cadherin resistance alleles was done using a modified protocol and PCR primers of that described by Morin et al. 2004 and Tabashnik et al. 2005a. Three sets of *P. gossypiella* adults from the Bt4R strain were screened for the presence of known *r* alleles. First, PCR was performed on DNA from adult *P. gossypiella* (n = 29) of the Bt4R strain that fed on Bt bolls and was the first generation of survivors on artificial diet containing 10 µg Cry1Ac toxin per ml diet. Second, DNA from Bt4R individuals (n = 19) maintained on 10 µg Cry1Ac per ml diet for ten generations was tested. Finally, Bt4R individuals (n = 20) were again tested for *r* alleles after 16 generations, with all but one generation of larvae reared on diet with 10 µg Cry1Ac per ml diet. DNA was extracted from *P. gossypiella* using PUREGENE DNA Isolation Kit from Gentra Systems (www.qiagen.com). PCR positive control reactions using DNA extracted *P. gossypiella* individuals from the diet-selected resistant strain (AZP-R) containing known *r* alleles (*r1r3* and *r2r3*) were performed as described by Morin et al. 2004.

### Reciprocal cross matings

To determine the effect of Cry1Ac resistance on mating, oviposition, larval survival and development of the Bt4R strain, male and female fourth instar larvae were separately reared on artificial diet containing 10 µg Cry1Ac/ml. Upon pupation, five Bt4R female pupae were paired with five WCRL male pupae and five WCRL female pupae were paired with five Bt4R male pupae and placed in cages. Tests were also performed on crosses with five Bt4R males paired with five Bt4R females and five WCRL males paired with five WCRL females. Eclosion occurred equally and timing was nearly synchronous for both strains. Adults were provided with oviposition substrates and a 10% sucrose solution. Eggs were collected after 10 days when >90% of oviposition should have occurred (Lingren et al. 1988). Fecundity and egg hatch from these crosses were compared to control crosses that consisted of adults obtained from WCRL larvae reared on artificial diet without Cry1Ac toxin and handled similarly in other respects. Numbers of eggs laid and percentage of eggs hatched were recorded. Adult females were dissected and examined for the presence of spermatophores to verify mating status. Mating and oviposition tests were replicated five times.

Oviposition and development of insects resulting from reciprocal crosses of Bt4R and WCRL were analyzed. Both the total number of eggs laid and egg hatch were examined. Approximately one half of the eggs were placed in containers with ∼ 2 g of either untreated diet or diet containing 10 µg Cry1Ac per ml diet. Eggs were incubated at 26.7°C (14:10 L:D) for 5 days and egg hatch was recorded. Larvae on diet were returned to 26.7°C for 21 days. Larval instar development and numbers of each on either untreated diet or on 10 µg Cry1Ac per ml diet were recorded. Egg and larval development experiments were replicated five times.

### Statistics

Data were analyzed by ANOVA using MSTAT (version 2.11) for comparisons between moth strains for differences in immature larval survival, fecundity, egg and larval mortalities. Percent mated females, spermatophore accrual, and oviposition were also analyzed pair wise by Mann-Whitney Rank Sum Test using SigmaStat (version 3.0). Mortality percentages were corrected for control mortalities using Abbott's formula (Abbot 1925), arcsine transformed, and analyzed by Holm-Sidak multiple comparison test using SigmaStat.

## Results

### Cry1Ac susceptibility

**Figure 1.**
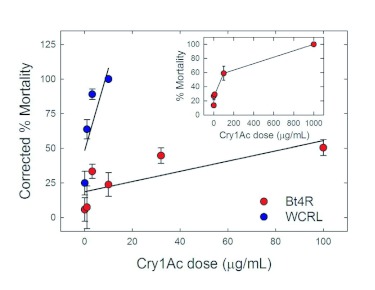
*Pectinophora gossypiella* dose-mortality response curves to Cry1Ac-incorporated artificial diet. Mean corrected % mortality plotted from 10–18 replications of five larvae per replicate. Insert shows mortality curve for Bt4R obtained from single larva bioassays with Cry1Ac concentrations of 1–1000 µg ml^-1^ and from which LC_50_ was estimated. Error bars are standard errors.

Larvae from the WCRL strain were more susceptible to Cry1Ac than the Bt4R strain (Figure 1 and Table 1). Mortality was significantly less for the Bt4R strain than for the WCRL strain (F = 4.69, df = 3, 110, *P* = 0.004). Mean LC_50_s for the WCRL and Bt4R strains were 0.254 µg/ml and 60.6 µg/ml, respectively (Table 1). Based on the LC_50_s, Bt4R was estimated to be 240-fold more resistant to Cry1Ac relative to WCRL. Other diet-selected strains of *P. gossypiella* had higher LC_50_ values and resistance ratios than Bt4R, including 700 µg/ml and 3,100-fold for AZP-R, 400 µg/ml and 1,700-fold for MOV97-R, and 120 µg/ml and 520-fold for SAF97-R, respectively (Tabashnik et al. 2002; Tabashnik et al. 2005a). The LC_50_ value for WCRL is comparable with that determined for another lab strain of *P. gossypiella* susceptible to Cry1Ac (LC_50_ = 0.23 µg/ml) (Tabashnik et al. 2005a).

**Table 1.**
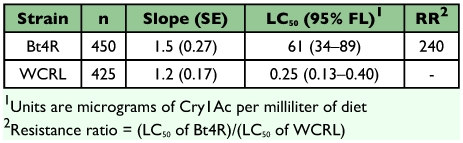
Responses to Cry1Ac of pink bollworm larvae resistant strain (Bt4R) selected on both Bt cotton and artificial diet and the unselected parent strain (WCRL).

### PCR analysis for known cadherin *r* alleles

Screening of Bt4R adults (n = 29 from F1, n = 19 from F10, and n = 20 from F16) for the presence of known *r* alleles failed to detect any of the previously identified cadherin *r* alleles. All individuals tested positive for the internal intron control, indicating that the DNA was intact at the cadherin locus in all samples. Likewise, DNA from individuals from diet-selected strains with known *r* alleles (*r1r3* and *r2r3*) was positively identified and genotyped. These results indicate that those Bt4R individuals selected on NuCOTN33B® Bt cotton bolls and 10 µg Cry1Ac per ml diet in the artificial diet bioassay did not possess *r1, r2*, or *r3* cadherin resistance alleles. It is not known whether these resistant *P. gossypiella* individuals contain additional mutations in the cadherin gene (i.e. *r4, r5*, etc.) or if the resistance is associated with other unknown resistance genes.

### Reciprocal cross mating, oviposition and egg hatch

Mating of males and females within strains (Bt4R X Bt4R and WCRL X WCRL) were not significantly different from individuals mated between strains (Bt4R X WCRL) (Table 2). Also, numbers of spermatophores found per female were not significantly different for the WCRL strain compared to females of the reciprocal pairings between strains or within the Bt4R strain. The total numbers of eggs laid, mean number of viable eggs, and mean number of viable eggs per mated female were reduced for mating pairs involving Bt4R strain moths. Specifically, there was a significant reduction in oviposition and mean numbers of viable eggs (total and per mated female) for the cross involving Bt4R females and WCRL males (Table 2). All crosses involving Bt4R showed reduced oviposition compared to mating within the WCRL strain. Like those reported by Carrière et al. (2001a, Carrière et al. 2001b), our results suggest a fitness cost is associated with resistance to Cry1Ac in Bt4R which impacts mating and oviposition. However, other factors such as moth age, length of time after emergence before mating occurs, inbreeding depression, and incomplete resistance may also contribute to the differences in oviposition.

**Table 2.**
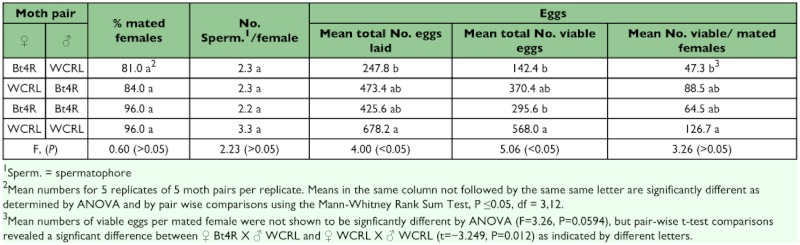
Mean percentages of mated females, spermatophores per female, and oviposition from crosses of WCRL and Bt4R moth pairs.

For larvae of WCRL and Bt4R reciprocal crosses feeding on untreated diet, the majority of the surviving progeny were 4^th^ instar larvae by 21 days (Table 3). Except for the Bt4R (♀) X Bt4R (♂) cross, the proportion of larvae that reached the 4^th^ instar when reared on artificial diet containing 10 µg Cry1Ac per ml diet by 21 days was significantly less than those larvae feeding on diet without Cry1Ac. Intrastrain crosses with Bt4R provided a clear advantage over crosses with the WCRL strain, with a significantly higher mean proportion of larvae reaching the 4^th^ instar and higher corrected percent survival. The corrected percent survival indicates an apparent disadvantage for progeny of Bt4R X WCRL cross with the Bt4R mother (0.74% adjusted survival) compared to the Bt4R father (8.9% adjusted survival) for 21 days survival on 10 µg Cry1Ac/ml diet. However, the mean proportion of progeny reaching 4^th^ instar larvae was not significantly different for Bt4R (♀) X WCRL (♂) versus WCRL (♀) X Bt4R (♂) indicating that the paternal sex linkage is not likely to be significant. Any cost associated with Bt4R (♀) X WCRL (♂) also did not affect oviposition, female mating, or spermatophore accrual (Table 2) or egg viability, as percentage of viable eggs obtained for all crosses from larvae reared on untreated artificial diet were not significantly different (Table 3).

## Discussion

The survival of F1 progeny from reciprocal crosses of resistant (Bt4R) and susceptible (WCRL) pink bollworm moths on 10 µg Cry1Ac per ml diet is in contrast to the results reported by Liu et al. (2001) and Tabashnik et al. (2002). These studies reported that dominance of Bt resistance was related to the concentration of Cry1Ac in artificial diets. Co-dominance occurred at low diet concentrations (0.1 µg/ml), partially recessive resistance at intermediate concentrations (1.0 µg/ml), and completely recessive (no survival) inheritance at high concentrations (>10 µg/ml). Although our studies were not designed to define the level of dominance of Cry1Ac resistance in Bt4R, some F1 progeny of the Bt4R (♀) X WCRL (♂) and WCRL (♀) X Bt4R (♂) crosses survived on artificial diet containing a discriminating concentration of Cry1Ac. This indicates some level of dominance in the resistance of Bt4R to Cry1Ac and differs from any of the diet-selected strains tested by Liu et al. (2001) and Tabashnik et al. (2002).

Studies of insect responses to Bt plants suggest that resistance to Bt toxins in diets or leaf dip bioassays does not always translate to the ability of the resultant strain to survive on their Bt host plant (Tabashnik et al. 2003). Larvae of resistant strains of European corn borer, *Ostrinia nubilalis* (Huang et al. 2002), Colorado potato beetle, *Leptinotarsa decemlineata* (Wierenga et al. 1996), and tobacco budworm, *Heliothis virescens* (Gould et al. 1995), are examples of resistant insects that do not survive on their Bt hosts. In contrast, resistant larvae of Diamondback moth, *Plutella xylostella* (Tabashnik et al. 1990), pink bollworm, and *Helicoverpa amigera* (Akhurst et al. 2003; Fan et al. 2000) have complete development on host plants expressing Cry1A toxins. Some selected insect strains may survive on Bt host plants and others may not because of greater exposure to toxin in transgenic plants, higher toxin concentration in plants, the presence of plant-toxin interactions, differences in production of active toxin in plants versus protein in diet bioassays, and/or differences in the selected resistance genes. Cry1Ac in cotton bolls has been reported by Liu et al. (2001) to be more toxic to pink bollworm larvae than artificial diet containing 10 µg toxin per ml diet. It is possible that one or more factors contribute to survival of Bt4R on Cry1Ac in diet bioassays but not on Bt cotton bolls. Selection for 42 generations on Bt cotton and additional selection of 16 generations on 10 µg Cry1Ac per ml diet did not produce larvae that survive on Bt cotton bolls. It is possible that more rigorous selection on Cry1Ac in subsequent generations of Bt4R may increase resistance to allow for survival on high concentrations of Cry1Ac and on Bt cotton. Resistance to Cry1Ac in *P. gossypiella* may evolve by selection of major or minor resistance genes (Groeters and Tabashnik 2000). Major resistance genes often favor selection of one or few loci that show rapid changes in frequency and provide high levels of resistance. It is likely that resistance to Cry1Ac in Bt4R is controlled by a minor gene or genes, as a major allele would likely be fixed under intense selection (Groeters and Tabashnik 2000). Evolution of resistance to Bt toxin may also be influenced by inherent differences in the toxin, such as protoxin activation status and/or toxin formulation as discussed by Anilkumar et al. (2008). More detailed studies are needed to determine the genetic basis for resistance to Cry1Ac in the Bt4R strain. Also, further studies are needed to determine differences in Cry1Ac expressed in Bt cotton bolls and/or other factors contributing to sustained susceptibility in Bt4R on plants versus toxin used in diet bioassays.

**Table 3.**
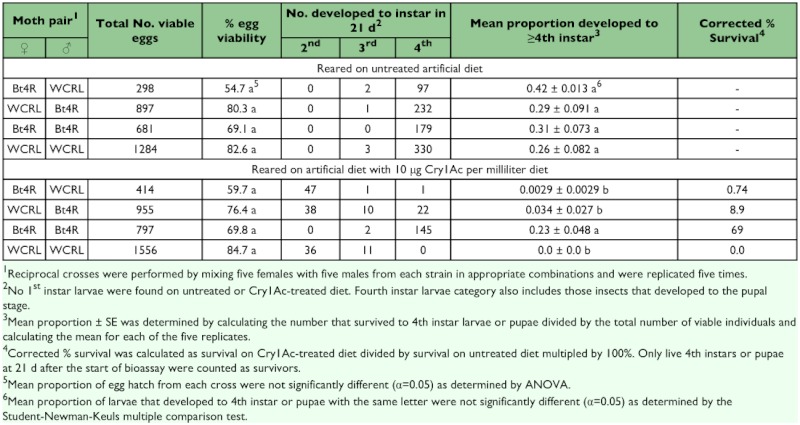
Larval survival, egg viability, and instar development of F1 progeny from WCRL and Bt4R reciprocal crosses.

Our results suggest that resistance to Cry1Ac found in Bt4R differs from diet-selected *P. gossypiella* strains. The pink bollworm strains of Liu et al. (2001) and Tabashnik et al. (2002) were selected for higher levels of resistance using increasing amounts of Cry1Ac toxin with increasing generations. Resistance in these strains was recessively inherited and genetically linked to mutations in a cadherin BtR gene (Morin et al. 2003; Tabashnik et al. 2004; Tabashnik et al. 2005a). We did not detect *r1, r2*, or *r3* resistance alleles in Bt4R. Therefore, Bt4R may differ from previous Cry1Ac-resistant strains either by a completely novel resistance mechanism or by unknown cadherin alleles not detected by allele-specific PCR. The fact that a unique selection regime (4 d on Bt plant vs. 21 d on Cry1Ac-treated diet) was used and because the apparent dominance of resistance differs from previously identified diet-selected resistant strains suggests that Bt4R employs a novel mechanism of resistance.

It is noteworthy that selection of resistance for 42 generations on Bt cotton bolls and 16 generations on Cry1Actreated artificial diet resulted in a resistant strain that is still unable to survive on Bt cotton plants. Field monitoring for *P. gossypiella* resistance to Bt cotton throughout the southwestern U.S. has not detected an increase in resistance allele frequency from 1997–2007 (Tabashnik et al. 2005b; Tabashnik personal communication) and PCR screening has yet to detect cadherin resistance alleles in the field (Tabashnik et al. 2006). This suggests that although *P. gossypiella* has the genetic potential to evolve resistance to Bt cotton, this has not yet occurred in the field and that resistance alleles that allow survival on Bt cotton are rare. Bioassays of field-collected *P. gossypiella* using Cry1Ac-treated diet estimated that the mean frequency of resistance alleles from 2001–2005 was 0.024 (Tabashnik et al. 2005b). If resistance is partially dominant as that found for Bt4R, such that some *rs* individuals survive in bioassays at 10 µg Cry1Ac per ml, the frequency of the dominant *r* allele would be lower than the stated estimate, with the exact estimate depending on how dominant the resistance is. Further studies are needed to identify genetic differences in *P. gossypiella* resistant strains, to define the mechanism of Bt resistance in Bt4R, and understand potential costs associated with resistance, which may improve our understanding of resistance mechanisms in lepidopterans and increase our ability to plan future resistance management tactics if it occurs in field populations.
